# The protection of chronic cerebral ischemia by Hua-Feng-Dan involves the modulation of hippocampal transcriptome and gut dysbiosis

**DOI:** 10.1007/s11011-026-02017-1

**Published:** 2026-11-09

**Authors:** Bo Liu, Bo Huang, Yunyan Xu, Jie Liu

**Affiliations:** Key Lab for Basic Pharmacology and Joint International Laboratory of Ethnomedicine of Ministry of Education, Zunyi Medical University, Zunyi, 563003 China

**Keywords:** Hua-Feng-Dan, Chronic cerebral hypoperfusion, Behavioral deficit, Histopathology, RNA-seq analysis, Ingenuity pathway analysis, Gut microbiota

## Abstract

The protective effects of Hua-Feng-Dan recipes against chronic cerebral hypoperfusion-induced ischemia were examined; RNA-seq and 16 S-rRNA-seq were used to explore the molecular mechanisms. Male Sprague-Dawley rats underwent unilateral common carotid artery ligation surgery to establish chronic cerebral hypoperfusion (CCH) models. Then, the CCH rats were treated with various Hua-Feng-Dan recipes in diet for 20 days. Finally, behavioral tests were performed, followed by histopathology. Hippocampal RNA was isolated for RNA-seq and RT-qPCR analyses; Colon bacterial DNA was isolated for 16 S rRNA-seq and bioinformatics. The CCH rats exhibited cognition deficits evidenced by Behavioral tests. The neurons in the hippocampus CA1 area were damaged. Hua-Feng-Dan recipes as Reduced_H (0.5 g/kg), Original, and Nonferment improved behavioral deficits and morphological changes, while Reduced_L (0.25 g/kg) was ineffective. RNA-seq revealed distinct gene expression pattern among groups. Ingenuity pathway analysis of differentially expressed genes revealed Hua-Feng-Dan recipes ameliorated Canonical pathways involved in cytosolic Ca^2+^ overload, disruption of IGF homeostasis and Neutrophil degranulation; attenuated CCH-activated Upstream regulators such as IL1B, IL6, TNF, IGF1, and glucose metabolism. CCH-decreased Firmicutes/Bacteroidetes ratio were recovered by Hua-Feng-Dan recipes. CCH-increased *Prevotellaceae* and decreased *Lachnospiraceae* were attenuated; CCH-increased ASV1 and decreased ASV39 were brought to normal after Hua-Feng-Dan treatments. Similar to the efficacy against CCH, Reduced_L was ineffective in transcriptome and gut microbiota analysis. Hua-Feng-Dan recipes (Reduced_H, Original, and Nonferment) are effective against CCH-induced chronic cerebral ischemia, while Reduced_L was ineffective. The mechanisms of the protection are related to transcriptome regulation in the hippocampus, and modulation of CCH-disrupted gut dysbiosis.

## Introduction

Hua-Feng-Dan has 300-year history in the treatment of brain diseases and is composed of “*yaomu*”, herbs, minerals, and others; The complete botanical names, batch numbers, and the proportions of each component can be found in Front Pharmacol (He et al. 2025; Liu et al. 2018b) and J Ethnopharmacology (Liu et al. 2018b; Han et al. 2025). Hua-Feng-Dan was patented as brain medicine in 2004 (CN02134139.7), and listed in the National Intangible Cultural Heritage in 2008. Pharmacological studies demonstrate its efficacy against acute cerebral ischemic stroke (Yuan et al. 2025; Shu et al. 2025; He et al. 2025), LPS plus Rotenone or MPTP-induced dopaminergic neuron loss (Hu et al. 2020; Chen et al. 2020) and PTZ-induced seizures (Li et al. 2026).

GC-MS identified 44 volatile components and 50 lipid soluble components in Hua-Feng-Dan (Liu et al. 2022). UPLC Q-TOF-MS/MS identified 54 novel components in Hua-Feng-Dan and detected 51 different components between ferment and nonferment Hua-Feng-Dan (Han et al. 2025). A recent Integrated UHPLC-Q-TOF/MS further identified 9 compounds in *yaomu*, which is important for fermented Hua-Feng-Dan in exerting hepatoprotective effects (Ma et al. 2026).

The protective effects of Hua-Feng-Dan against acute cerebral stroke simulated by middle cerebral artery occlusion (MCAO) have been reported (He et al. 2025; Han et al. 2025; Yuan et al. 2025). However, the effects of Hua-Feng-Dan on chronic cerebral ischemia were uncovered. Chronic cerebral hypoperfusion (CCH) is a progressive cerebrovascular disorder primarily attributable to cerebral arteriosclerosis and other vascular risk factors, resulting in sustained chronic cerebral ischemia(Bauer et al. 2015; Ma et al. 2025b). Clinically, it manifests as progressive cognitive decline and is associated with vascular dementia (Duncombe et al. 2017; Ma et al. 2025b) and Alzheimer’s disease (de la Torre 2021). The common carotid artery occlusion model is a well-established experimental paradigm for simulating CCH (Liu et al. 2018a). To enhance model feasibility and animal welfare while preserving construct validity, we employed unilateral common carotid artery ligation as a refined, lower-mortality alternative for establishing the rat model of CCH (Yang et al. 2016; Ma et al. 2025b).

For safety consideration, both cinnabar (αHgS) and realgar are reduced from 10% (termed “Original”) to 3% (termed “Reduced” or “Reduced_H”) (Chen et al. 2020; Han et al. 2025; Li et al. 2026). Moreover, the “*Yaomu*” plays a significant role in the pharmacological effects of Hua-Feng-Dan (Han et al. 2025). The 6 ingredients of *Yaomu* were added raw without 3-month fermentation termed “Nonferment” (Li et al. 2026).

In this study, we examined the effects of 20-day Hua-Feng-Dan recipes (Reduced, Original, and Nonferment) treatment in CCH rats, and used hippocampal RNA-seq and gut microbiota 16 S rRNA-seq to analyze the molecular mechanisms of the protection. Firstly, the effects of Hua-Feng-Dan on cognitive function of rats were observed, and secondly, neuronal damage was evaluated by HE staining. Finally, hippocampal RNA was extracted for RNA-seq, and gut bacterial DNA was extracted for 16 S rRNA-seq. Comprehensive bioinformatics were performed to explore the molecular targets. The results clearly demonstrated the multicomponent and multitarget nature of Hua-Feng-Dan as the ethnomedicine in the treatment of chronic cerebral ischemia.

## Materials and methods

### Animals

Two-month-old male Sprague-Dawley (SD) rats (290 ~ 340 g) were bought from the Beijing Vital River Laboratory Animal Technology Co., Ltd. (SPF grade, Certificate No. SCXK2021-0006). All the rats were allowed to undergo acclimatization feeding for one week before experimentation. Rats were maintained in a 12 h light/dark cycle at 22–25 ℃ and relative humidity 50%, and food and water *ad libitum*. Measures were designed to reduce the number of rats and minimize their suffering. The animal experiment procedures were in accordance with the Chinese Guidelines for the Care and Use of Animal, and the study protocol was proved by the Institutional Animal Use and Care Committee with Ethics No.: [2020]2-142.

### Surgery

To enhance model feasibility and animal welfare while preserving construct validity, we employed unilateral common carotid artery ligation as a refined, lower-mortality alternative for establishing the rat model of CCH (Yang et al. 2016; Ma et al. 2025b). The CCH model was established according to the methods previously reported (Kimura et al. 2025). After administering 2% pentobarbital sodium (3 ml/kg, i.p., Sigma, P3761) for anesthesia, the left common carotid artery of the rat was isolated through a median incision in the neck, then ligation is performed with surgical silk threads. This model can reduce cerebral blood flow to 70%-75% of the baseline (Kim et al. 2020). Compared with the rat model of bilateral common carotid artery ligation, the injury degree is relatively small. It also induces atrophy of the hippocampus and enlargement of the lateral ventricles (Kimura et al. 2025), as reviewed in our recent publication (Ma et al. 2025b). The procedure was carried out on a heating pad to maintain the rat’s body temperature at 37.5 ± 0.5 ℃ until anesthesia was recovered. In the Cont group, the same surgical exposure was performed as a sham group, and no ligation was carried out. The incision site was disinfected with iodophor, and the wound was closed with absorbable sutures.

### Experimental design

**Fig. 1:**
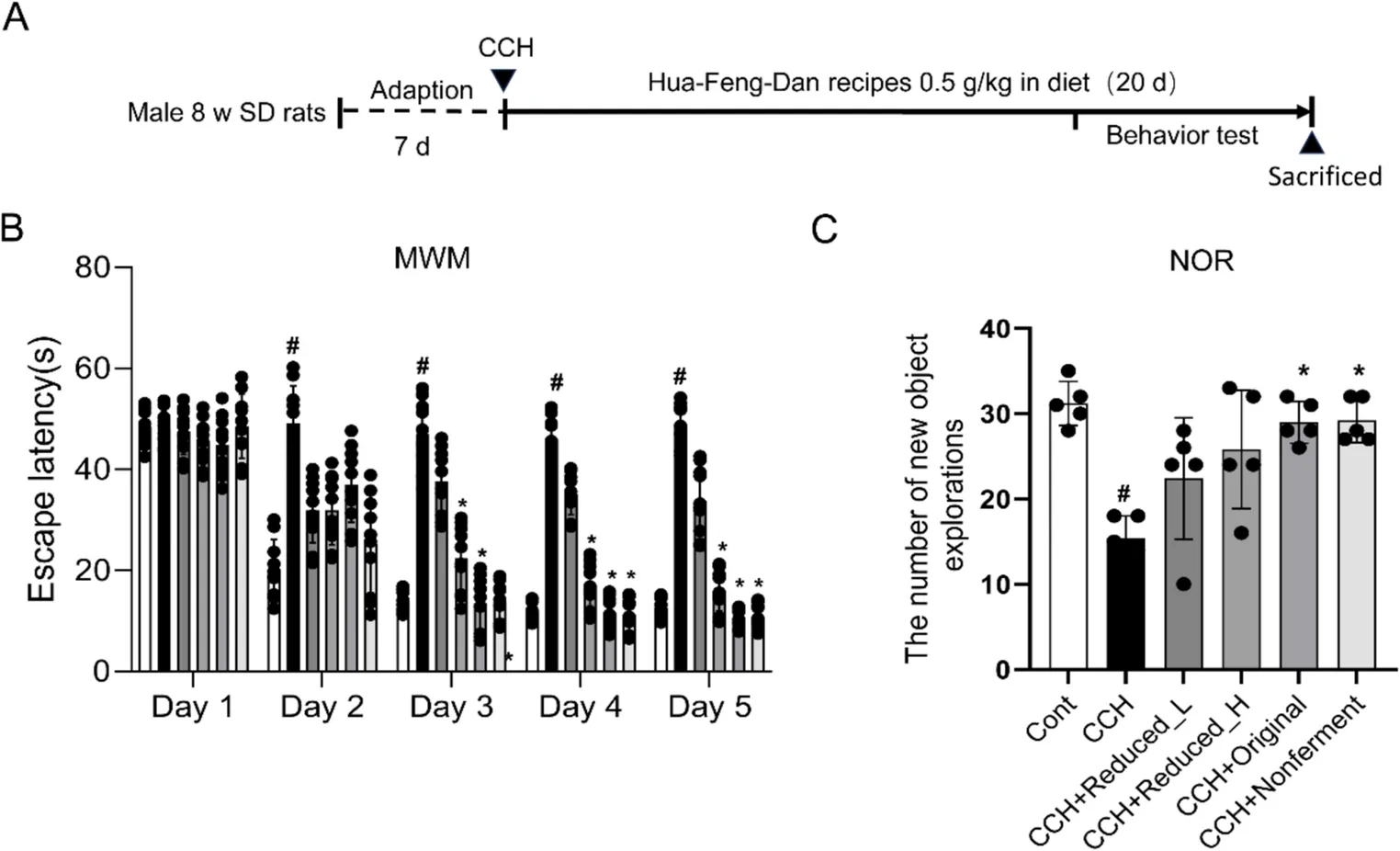
**The Morris water maze (MWM) and Novel object recognition test (NOR) demonstrated that Hua-Feng-Dan could alleviate the cognitive impairment in CCH rats.** (**A**) Experimental overview chart. (**B**) The results of Morris water maze(MWM). Repeated measures multivariate analysis of variance was used to assess the results of MWM (Values are presented as mean ± SD, *n* = 10). (**C**) The results of the Novel object recognition test (NOR). (Data were subjected to one-way analysis of variance (ANOVA), followed by Dunn’s multiple range tests. Data are presented as mean **±** SD, *n* = 5). #*p* < 0.05 vs. Cont group, *p < 0.05 vs. CCH group

Rats were randomly divided to Cont, CCH, CCH+Reduced_L (containing 3% cinnabar and 3% realgar, 0.25 g/kg), CCH+Reduced_H (containing 3% cinnabar and 3% realgar, 0.5 g/kg), CCH+Original (containing 10% cinnabar and 10% realgar, 0.5 g/kg), CCH+Nonferment (the Guide Drug “Yaomu” was not fermented for 3 months, 0.5 g/kg) groups, 10 rats each group. The day after CCH surgery, The Cont and CCH group with normal diet, and other groups were given various Hua-Feng-Dan recipes (Guizhou Wansheng Pharmaceutical Co., Ltd., China) via diet based on the previously mentioned clinical dosage *(*Li et al. 2026). According to feed consumption, the approximate doses were 0.5 g/kg Hua-Feng-Dan for CCH+Reduced_H, Original, Nonferment groups, and 0.25 g/kg Hua-Feng-Dan for CCH+Reduced_L group to assess dose-dependent effects. The Hua-Feng-Dan dosage and administration method was on the basis of our previous publications *(*Li et al. 2026; Hu et al. 2020; Chen et al. 2020). All diets were prepared by Jiangsu Xietong Biology (Nanjing, China) (Li et al. 2026). Under these doses with 20-30-day of treatment with Hua-Feng-Dan, the animal body weight and general health parameters are within the normal range in rats (Chen et al. 2020) and in mice (Hu et al. 2020; Li et al. 2026). The Cont and CCH groups were given normal diet. All treatments lasted for 20 days. Behavioral experiments were performed 15–20 days of treatments (Fig. 1A). After the behavioral tests, the rats were euthanized. Brain tissues from 4 rats in each group were used for pathological examination. The hippocampus from the remaining 6 rats was isolated and frozen at − 80 °C for RT-qPCR analysis, and half of the hippocampus from 3 of these rats was randomly selected for RNA-seq analysis. Colon contents were collected for bacterial DNA extraction followed by 16 S rRNA-seq.

### Behavioral tests

#### Morris water maze (MWM)

MWM test was used to evaluate the spatial learning and memory of rats. Biefly, in the circular heated water pool (diameter 120 cm, height 50 cm), water temperature was maintained in 24–26 °C, and powdered milk (1 kg) was added to make the water opaque. To ensure the accurate spatial positioning of the mice, all external visual cues around the pool remained unchanged. A platform was placed 2 cm underwater in the fourth quadrant and the escape platform location remained constant throughout the acquisition training period. The experiment spanned over 5 consecutive training days. Each animal faced the wall of the pool was placed in the pool, and the time (escape latency) for each rat to swim from the starting position (four different quadrant starting positions) to the submerged platform was recorded. If an animal fails to reach the hidden platform within the specified time, guide it to stay on the platform for 15 s. In this case, the escape latency time was recorded as 120 s. The mice were trained four times daily, with 60 s rest between each training and the data are the average of the four trials. The behavioral performance was recorded by a computer-based video tracking system and analyzed using MT-200 image analysis software (from Taimeng Company in Chengdu, China) (Liu et al. 2018a).

#### Novel object recognition test (NOR)

Learning and memory abilities were also tested by NOR. The NOR test is composed of a reaction box (length 72 cm, width 72 cm, height 50 cm), behavioral analysis software (Topscan, CleverSys, Inc., Reston, VA, USA), and camera equipment. The first day, each rat was placed in the reaction box to adapt to the environment for 10 min. The second day, two yellow cylinders 3 cm in diameter were located in the two corners of the reaction chamber, and rats were placed into the reaction chamber facing the wall and allowed to move freely for 5 min. The third day, one yellow cylinder was replaced by a new blue cone. All of the rats were free to explore 5 min in the chamber. The distance between the nose of rat and the novel blue cone within 2 cm was defined as exploratory behavior. The exploration time of new blue cone were recorded (Liu et al. 2020).

### Hematoxylin-eosin (HE) staining

After the behavioral test, the rats were anesthetized and perfused with cold NS solution and 4% paraformaldehyde (MedChemExpress, HY-Y0333, USA) through the heart. Then, the rat brain was removed and immersed in 4% paraformaldehyde solution (MedChemExpress, HY-Y0333, USA) at 4 ℃ for 24 h. The hippocampus coronal sections behind optic chiasma were selected and fixed. The continuous Sects.  (4–5 μm) were stained with hematoxylin-eosin (MedChemExpress, HY-K0315, USA), and then the morphology of neurons in hippocampus CA1 was observed under optical microscope (Leica Microsystems Ltd., Wetzlar, Germany) (Liu et al. 2024a) and quantified with Image J as described previously (Liu et al. 2023).

### Hippocampus RNA isolation and sequencing

The total RNA of the hippocampus was extracted using Trizol reagent (Takara, 9108, Japan). RNA quality and quantity were detected using Nanodrop, gel electrophoresis and Agilent 2100 (RIN > 7). Oligodeoxynucleotide beads and poly A were used to enrich mRNA. Fragmented mRNA samples (300 bp) were reverse transcribed with random xamers and reverse transcriptase to produce cDNA. The generated single-stranded cDNA reacts with RNase H enzyme (Takara Biotechnology Co., Ltd., 2130B, USA), DNA polymerase I (Takara Biotechnology Co., Ltd.,EP0041, USA) and dNTPs (Takara Biotechnology Co., Ltd., 4025, USA) to produce double-stranded cDNA. After purification, the double-stranded cDNA was subjected to End Repair, addition of a tail and adaptor. Then, RNA sequencing was performed using the Illumina NovaSeq 6000 platform. The successfully constructed libraries were sequenced and analyzed by Majorbio Biotechnology Co., Ltd. (Shanghai, China) (Liu et al. 2024a).

### Principal component analysis (PCA)

Partek Flow (Partek Inc., St. Louis, MO) was used to perform PCA. Import the total original gene count of RNA-seq (18 samples, 30,563 per sample) into the Partek Flow Server, including the Cont, CCH, CCH+Reduced_L, CCH+Reduced_H, CCH+Original, CCH+Nonferment groups (*n* = 3), respectively. PCA images were generated by visualizing the gene expression patterns among groups (Liu et al. 2024a).

### **Differentially expressed genes (DEGs) analysis**

The DESeq2 method is used to analyze RNA-Seq data compared with the Cont group. The criteria for DEGs were set at *p* < 0.05.

### Reverse transcription-quantitative polymerase chain reaction (RT-qPCR)

Total RNA was quantified at 260/280 nm and reverse transcribed by Primerscript RT kit (Takara Biotechnology Co., Ltd., RR037A, China). Primer 3 was used to design the primers, and the primers were synthesized by Sangon Biotech (Shanghai, China) (*Supplementary Table 1*). The RT-qPCR reaction was carried out with a Bio-Rad CFX96 real-time PCR machine using iTaq Universal SYBR Green Mix (Bio-Rad, 1725121, USA) as described (Li et al. 2026).

### Ingenuity pathway analysis (IPA)

DEGs were uploaded into Ingenuity Pathway Analysis (IPA) (Qiagen, Redwood City, California, United States), and the Canonical pathways and Upstream regulators were analyzed with Z-scores (Li et al. 2026).

### **Microbial 16 S rRNA sequencing**

Colon contents were collected and stored at -80 °C. Bacterial DNA was extracted using a DNA kit (Solarbio, R1200, China) according to the manufacturer’s instructions. The quality and concentration of the DNA were determined using the NanoDrop ND-2000 (Thermo Fisher Scientific Inc., USA), ensuring a ratio of 260/280 > 1.8 and a concentration > 20 ng/µL. The microbiome analysis included 4 biological replicates (individual animal samples) per group. Sample processing and library sequencing were performed in a balanced manner across all experimental groups to minimize batch effects. Samples were then sent to Shanghai Majorbio Biopharmaceutical Technology Co., Ltd. (Shanghai, China) for 16 S rRNA-seq and bioinformatic analysis (Li et al. 2026).

### Statistical analysis

Each animal represented one independent biological replicate (n). Repeated measures multivariate analysis of variance was used to assess the results of MWM. Differentially expressed genes (DEGs) from RNA-Seq were analyzed by DeSeq2 using Padj ≤ 0.05 compared to the Cont group (p-value after Benjamini & Hochberg correction). Other data were subjected to one‑way ANOVA when homogeneity of variances was assumed, with Tukey’s HSD for post‑hoc pairwise comparisons. If the assumption was not met, the nonparametric Kruskal–Wallis test was performed, followed by Dunn’s test with Bonferroni correction for multiple pairwise comparisons. The data were expressed as mean ± SD. Statistical significance was *p* < 0.05.

## Result

### The effect of Hua-Feng-Dan on cognitive impairment in CCH rats

In the MWM, starting from the third day, CCH rats exhibited longer escape latency compared with the Cont rats (*p* < 0.05), and there was no obvious downward trend in the model group. Compared with the CCH group, the escape latency of the rats in the CCH+Reduced_H, CCH+Original, CCH+Nonferment groups decreased significantly with the increase in training times. This suggests that the CCH model was successfully established, and the rats in the CCH group exhibited obvious learning and memory impairment. Apart from Reduced_L treatment, all the other compound Hua-Feng-Dan preparations have a promoting effect on the spatial learning and memory of CCH rats (Fig. 1B).

In the NOR experiment, the number of new object explorations in the CCH group was significantly lower than that in the Cont group. The exploration attempts in the administration group were higher than those in the CCH group, and there were significant differences between the CCH+Original, CCH+Nonferment and the CCH group, suggesting that after administering different formulations of Hua-Feng-Dan, the learning and memory of CCH rats improved, especially the Original and the Nonferment Hua-Feng-Dan (Fig. 1C).

### The influence of Hua-Feng-Dan on the HE staining of rat hippocampus tissue

**Fig. 2:**
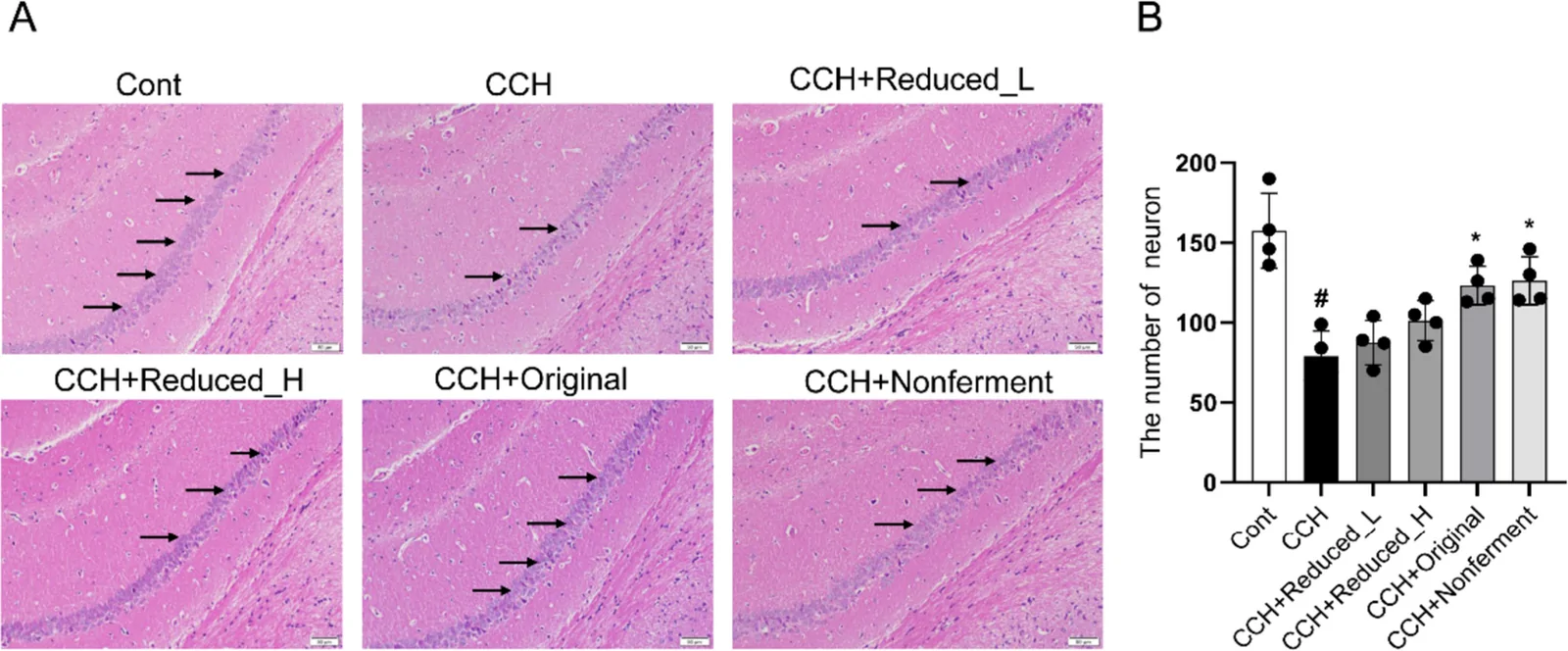
**HE staining demonstrated that Hua-Feng-Dan improved neuronal damage in the rat hippocampus CA1 region**. (**A**) The neurons in the hippocampal CA1 region. The black arrows highlight the normal-shaped neurons in the CA1 area of the hippocampus (Light microscopy magnification 200×, scale bar = 50 μm, *n* = 4); (**B**) Quantitation of neurons in the hippocampal CA1 region. Data were subjected to one-way analysis of variance (ANOVA), followed by Dunn’s multiple range tests. Data are presented as mean ± SD, *n* = 4, #*p* < 0.05 vs. Cont group, *p < 0.05 vs. CCH group

The HE staining results showed that the neurons in the CA1 region of the hippocampus in the Cont group were arranged neatly, with clear edges, regular shapes, uniform cytoplasmic staining, and clear cell nuclei. However, in the CCH group, the cytoplasmic staining of the cells in the hippocampus CA1 region was uneven, the cell nuclei were shrunken and stained darker, and the neurons arrangement was severely disordered and sparse. Compared with the CCH group, the hippocampal neurons in the different formulations of Hua-Feng-Dan administration groups were arranged more neatly, the degree of neuron damage was reduced and the cell nuclei became clearer (Fig. 2A). Moreover, Hua-Feng-Dan administration increased the number of neurons in the hippocampus CA1 region, especially in the Original and the Nonferment groups (Fig. 2B).

### Gene expression pattern analysis and differentially expressed gene analysis

**Fig. 3:**
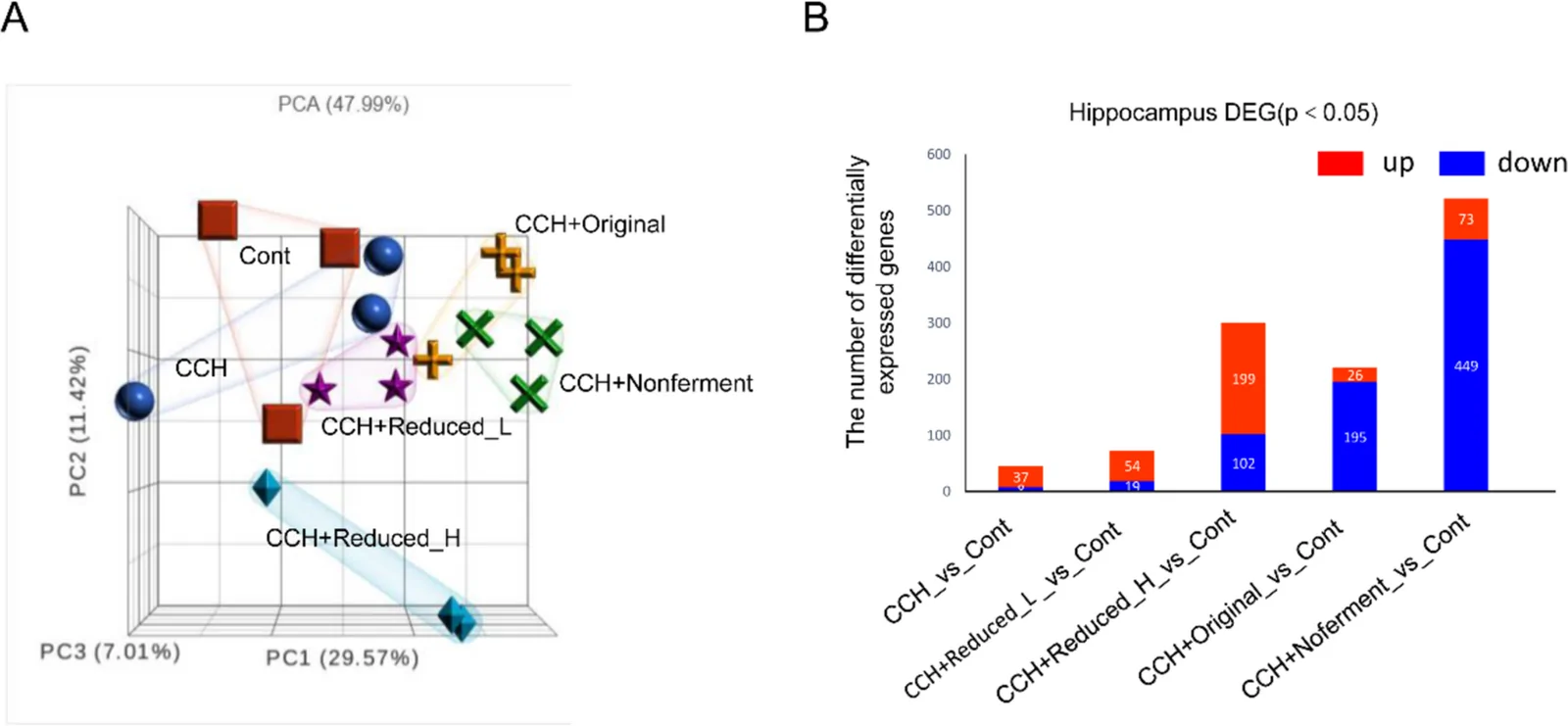
**Principal component analysis (PCA) and differentially expressed genes (DEGs) after Hua-Feng-Dan treatment.** (A) PCA of all 30,563 counts/sample shows distinct patterns among groups. Each group has been outlined in different colors. (B) Differentially expressed genes compared to Cont with upregulation in red color and downregulation in blue color. The differentially expressed genes were analyzed via the DESeq2 as Padj ≤ 0.05 compared to Cont group (*n* = 3). Red represents upregulation and blue represents downregulation

Total RNA from the hippocampus was extracted for RNA sequencing (Illumina HiSeq platform). FPKM (Fragments per kilobase of exon per million fragments mapped) obtained from RNA-Seq were processed to raw gene counts (30563/sample) and raw data were uploaded into Partek Flow for Principal component analysis (PCA). There were 18 individual samples and 6 groups (three samples/group). The PCA value is 47.99%, with PC1 = 29.57%, PC2 = 11.42%, and PC3 = 7.01%. The distributions of the CCH+Reduced_L, CCH+Reduced_H, CCH+Original, CCH+Nonferment groups were apparently separated from the CCH group, especially for CCH+Reduced_H, CCH+Original, and CCH+Nonferment groups (Fig. 3A).

The differentially expressed genes (DEGs) were analyzed via the DESeq2 method compared to Cont group (*p* < 0.05). CCH group resulted in 45 DEGs (37 up and 8 down); CCH+Reduced_L group resulted in 73 DEGs (54 up and 19 down), CCH+Reduced_H group resulted in 301 DEGs (199 up and 102 down), CCH+Original group resulted in 221 DEGs (26 up and 195 down), CCH+Nonferment groups resulted in 521 DEGs (73 up and 449 down) (Fig. 3B). It is obvious that compared with the Cont. group, the number of DEGs in the CCH group was the lowest, while the CCH+Reduced_H group had more up-regulated DEGs and the CCH+Nonferment had more down-regulated DEGs. The 2-D clusters of DEGs can be found in *Supplementary Fig. 1*.

### IPA of Canonical pathways and Upstream regulators

**Fig. 4:**
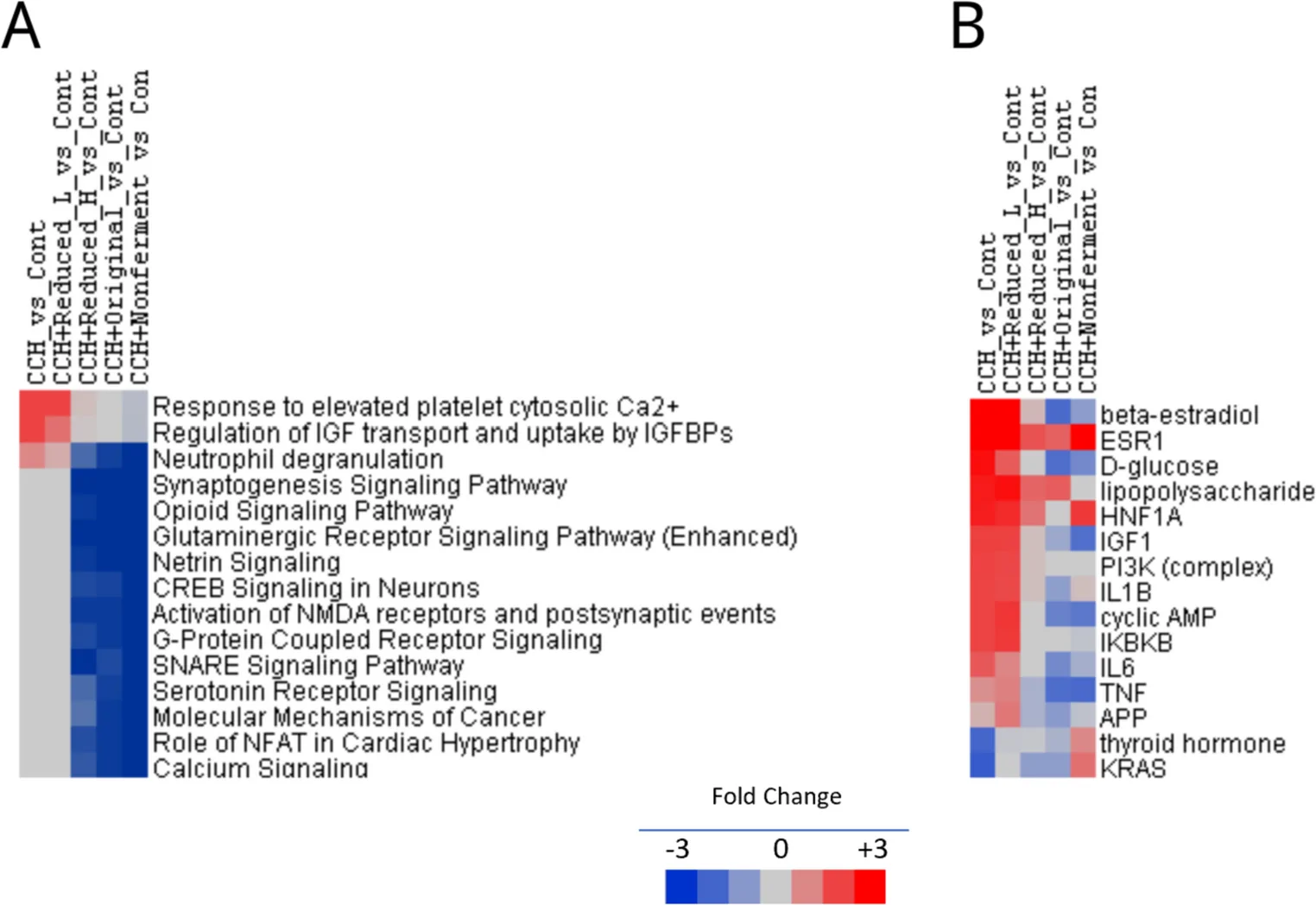
**Ingenuity Pathways Analysis (IPA) of Canonical Pathways and Upstream regulators after Hua-Feng-Dan treatment.** (**A**) Selected 15 canonical pathways from Ingenuity Pathways Analysis (IPA) of Canonical Pathways. (**B**) Selected 15 Upstream regulators from Ingenuity Pathways Analysis (IPA) of Upstream regulators. Compared to Cont group, the upregulation is shown in red color; the downregulation in blue color; and blank indicates no change based on Z-scores

Figure 4A shows the selected Canonical pathways, including upregulated pathways “Response to elevated platelet cytosolic Ca^2+^”, “Regulation of IGF transport and uptake by IGFBPs”, and “Neutrophil degranulation” in CCH group (red in color). Fortunately, the Reduced_H, Original and the Nonferment Hua-Feng-Dan reversed these pathways. The pathway of “Synaptogenesis Signaling Pathway”, “Netrin Signaling”, “CREB Signaling in Neurons” were decreased from normal level after Reduced_H, Original, and Nonferment treatment (blue in color) (Fig. 4A).

As shown in Fig. 4B, CCH_vs_Cont had top 15 upstream regulators, including 13 increases. The results show that the presence of the upstream regulators were closely related to inflammation (such as lipopolysaccharide, IL1B, IKBKB, IL6, TNF) and metabolism (beta-estradiol, IGF1, D-glucose, PI3K, APP). The groups of CCH+Reduced_H, CCH+Original and CCH+Nonferment attenuated these increases or changed these regulators to the opposite direction, while CCH+Reduced_L was not quite different from CCH in Fig. 4A and B.

### RT-qPCR analysis of genes related to inflammation

**Fig. 5:**
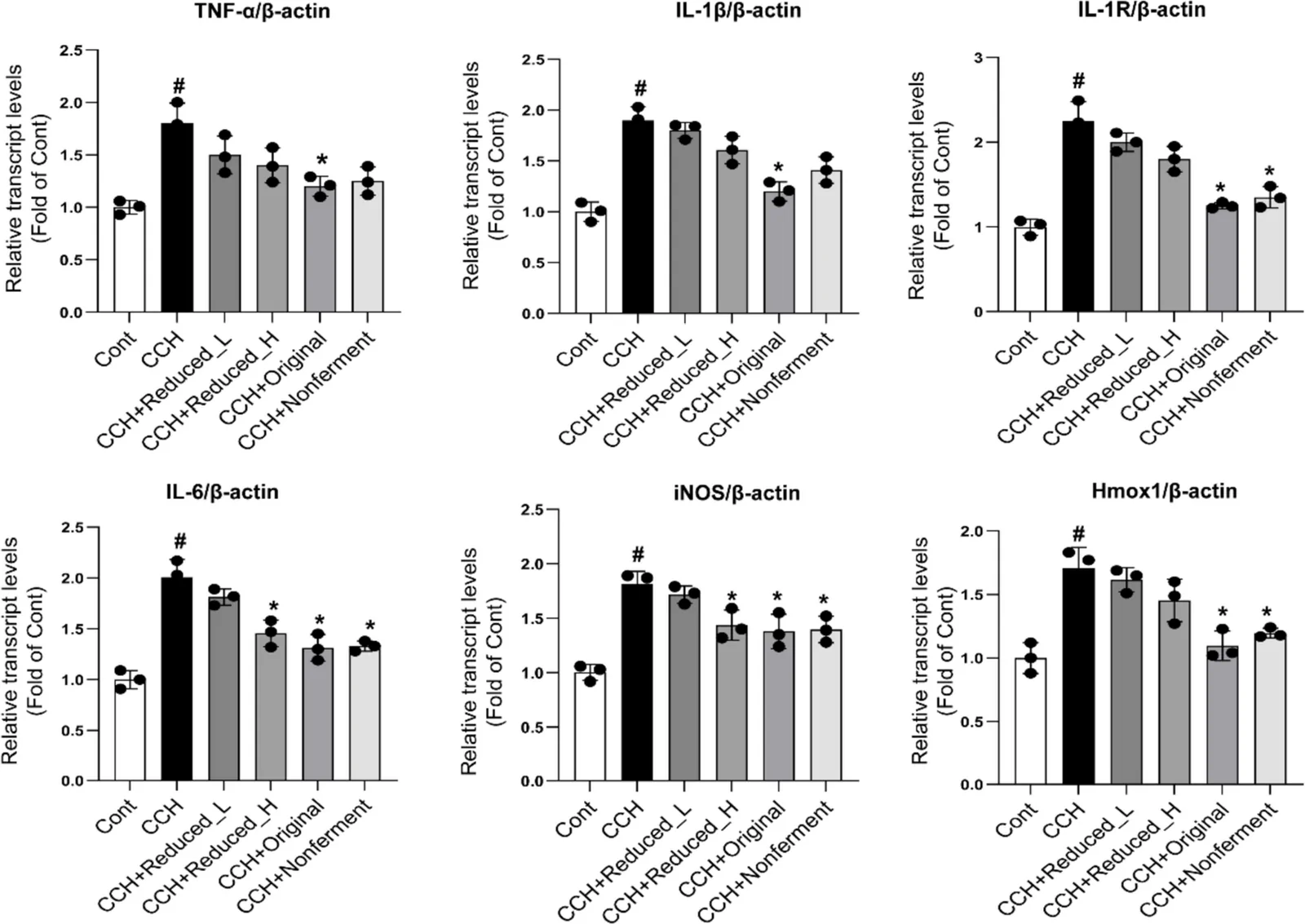
**RT-qPCR analysis of genes related to inflammation (TNF-α**,** IL-1β**,** IL-1R**,** IL-6**,** iNOS and Hmox1) after Hua-Feng-Dan treatment.** Data were subjected to one-way analysis of variance (ANOVA), followed by Dunn’s multiple range tests. Data are presented as mean ± SD, *n* = 6, #*p* < 0.05 vs. Cont group, *p < 0.05 vs. CCH group

Based on the results of IPA, the selected genes related to inflammation were verified through RT-qPCR, as shown in Fig. 5. Compared with the Cont group, TNF-α, IL-1β, IL-1R, IL-6, iNOS and Hmox1 expressions were increased in CCH rats, and most of different formulations of Hua-Feng-Dan treatments prevented these increases, especially the Reduced_H, Original and the Nonferment Hua-Feng-Dan, but were less effective after Reduced_L treatment (Fig. 5).

### Heatmap of altered genes and RT-qPCR analysis of selected DEGs

**Fig. 6:**
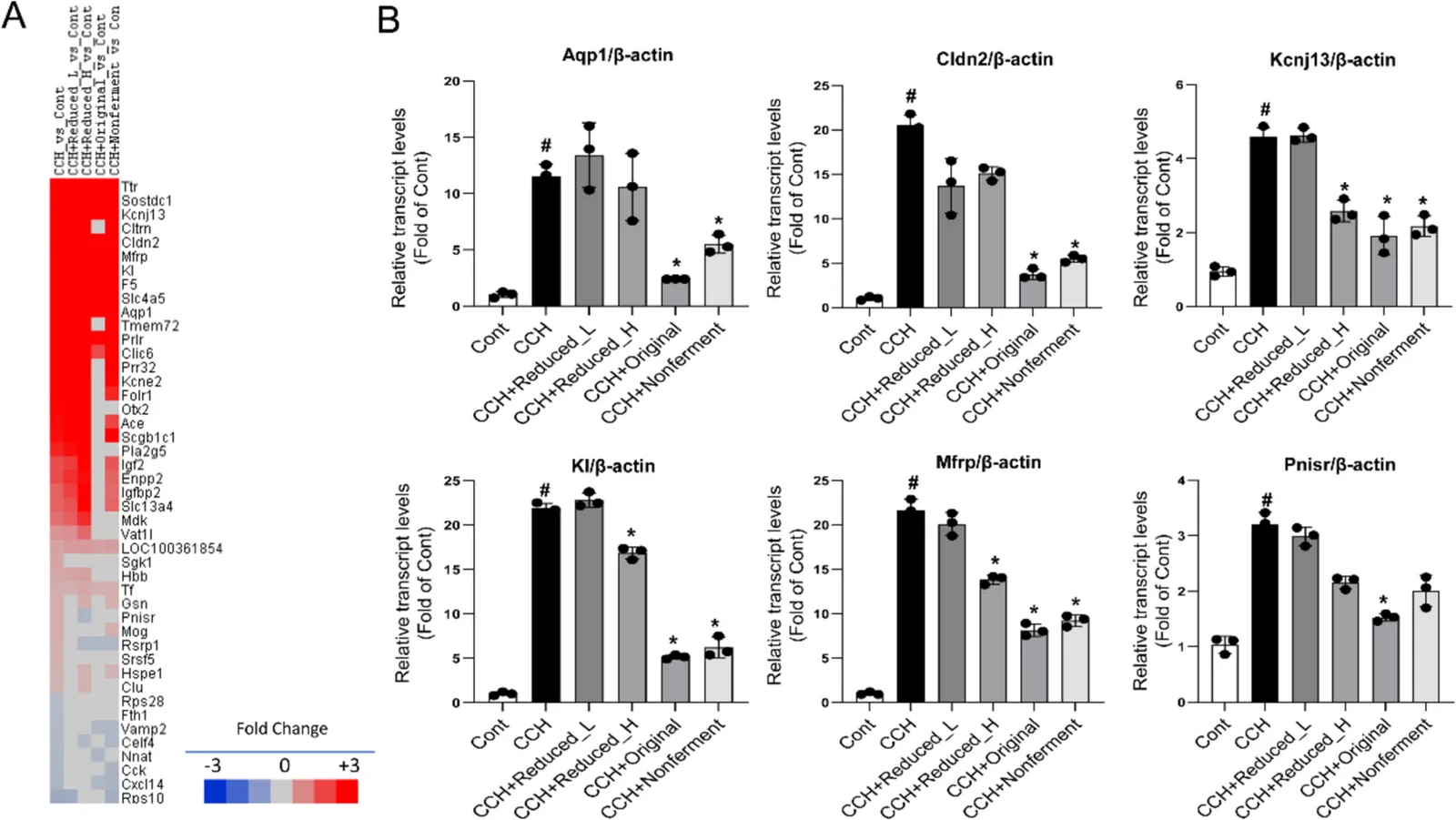
**Differentially expressed genes heatmap and RT-qPCR analysis of selected differentially expressed genes (DEGs) after Hua-Feng-Dan treatment.** (**A**) Heatmap of altered genes after Hua-Feng-Dan treatment. Upregulation shows in red color, downregulation in blue color, and blank indicates no change (compared to Cont group). (**B**) RT-qPCR analysis of selected differentially expressed genes (DEGs). Data were subjected to one-way analysis of variance (ANOVA), followed by Dunn’s multiple range tests. Data are presented as mean ± SD, *n* = 6, #*p* < 0.05 vs. Cont group, *p < 0.05 vs. CCH group

The heatmap of DEGs among groups based on CCH is shown in Fig. 6. Hua-Feng-Dan treatments attenuated aberrant gene expressions in CCH rats. For example, the increased gene expression in CCH rats was abolished following Hua-Feng-Dan treatment, including overexpression of Aqp1, Cldn2, Kcnj13, KI, Mfrp and Pnisr (Fig. 6A). Based on the heatmap, the selected 6 DEGs were verified by RT-qPCR (Fig. 6B). Compared with the Cont group, the expressions of Aqp1, Cldn2, Kcnj13, KI, Mfrp and Pnisr were increased in CCH rats, and most of different Hua-Feng-Dan formulations treatments prevented such increases, especially the CCH+Reduced_H, Original and the Nonferment Hua-Feng-Dan, but were less effective after CCH+Reduced_L treatment (Fig. 6).

### The effect of Hua-Feng-Dan on gut microbial α-diversity in CCH rats

**Fig. 7:**
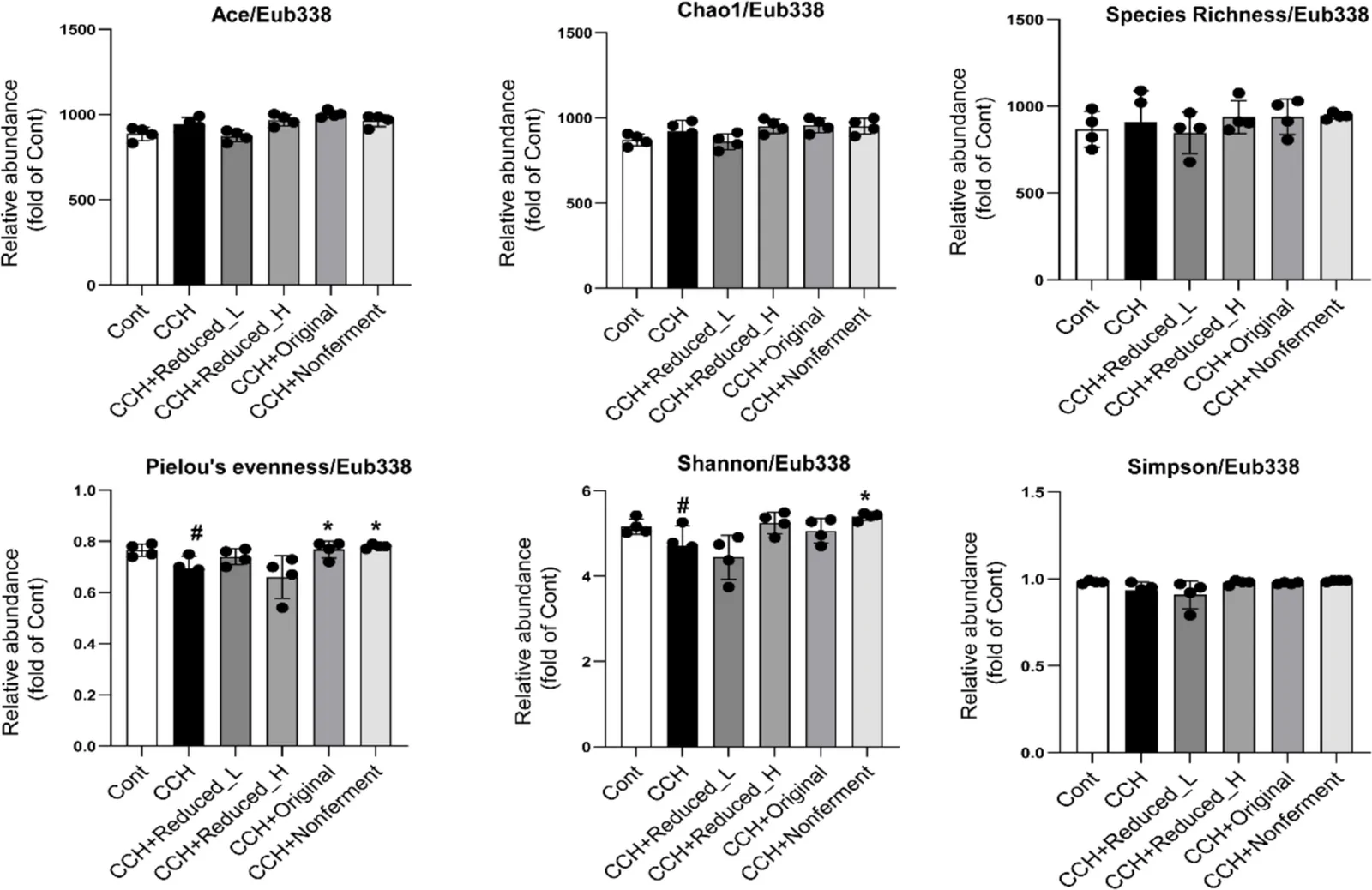
**Comparison of gut microbial diversity after Hua-Feng-Dan treatment.** α-diversity indices including observed Ace, Chao1, Special Richness, Pielou’s evenness, Shannon, and Simpson indices. Data were subjected to one-way analysis of variance (ANOVA), followed by Dunn’s multiple range tests. Data are presented as m mean ± SD, *n* = 4, #*p* < 0.05 vs. Cont group, *p < 0.05 vs. CCH group

Evidence indicates that the gut-microbiota-brain axis is closely related to brain disorders, including chronic cerebral hypoperfusion-induced cerebral ischemia (Klepinowski et al. 2024; Li et al. 2025). The composition of the gut microbiome and parameters of α-diversity are important to evaluate drug effects in the CCH model (Mao et al. 2024). α-diversity is a general term for metrics that describe the species richness (Ace, Chao1, Special Richness), evenness (Pielou’s evenness), or diversity (Shannon, Simpson) within the sample. It is obvious that the Ace, Chao1 and Special Richness indices did not show significant differences among groups, suggesting that the richness of the microbial communities remained relatively unaffected. The Pielou’s evenness were decreased in CCH rats, and the Original and Nonferment Hua-Feng-Dan treatment reversed the downward trend. The Shannon and Simpson showed a downward trend in the CCH goup, the Reduced_H, Original and the Nonferment Hua-Feng-Dan prevented such in decreases, suggesting that the evenness and comprehensive diversity were affected by Reduced_H, Original and the Nonferment Hua-Feng-Dan treatment (Fig. 7).

### The effect of Hua-Feng-Dan on the phylum, family level and ASV abundance of gut microbial in CCH rats

**Fig. 8:**
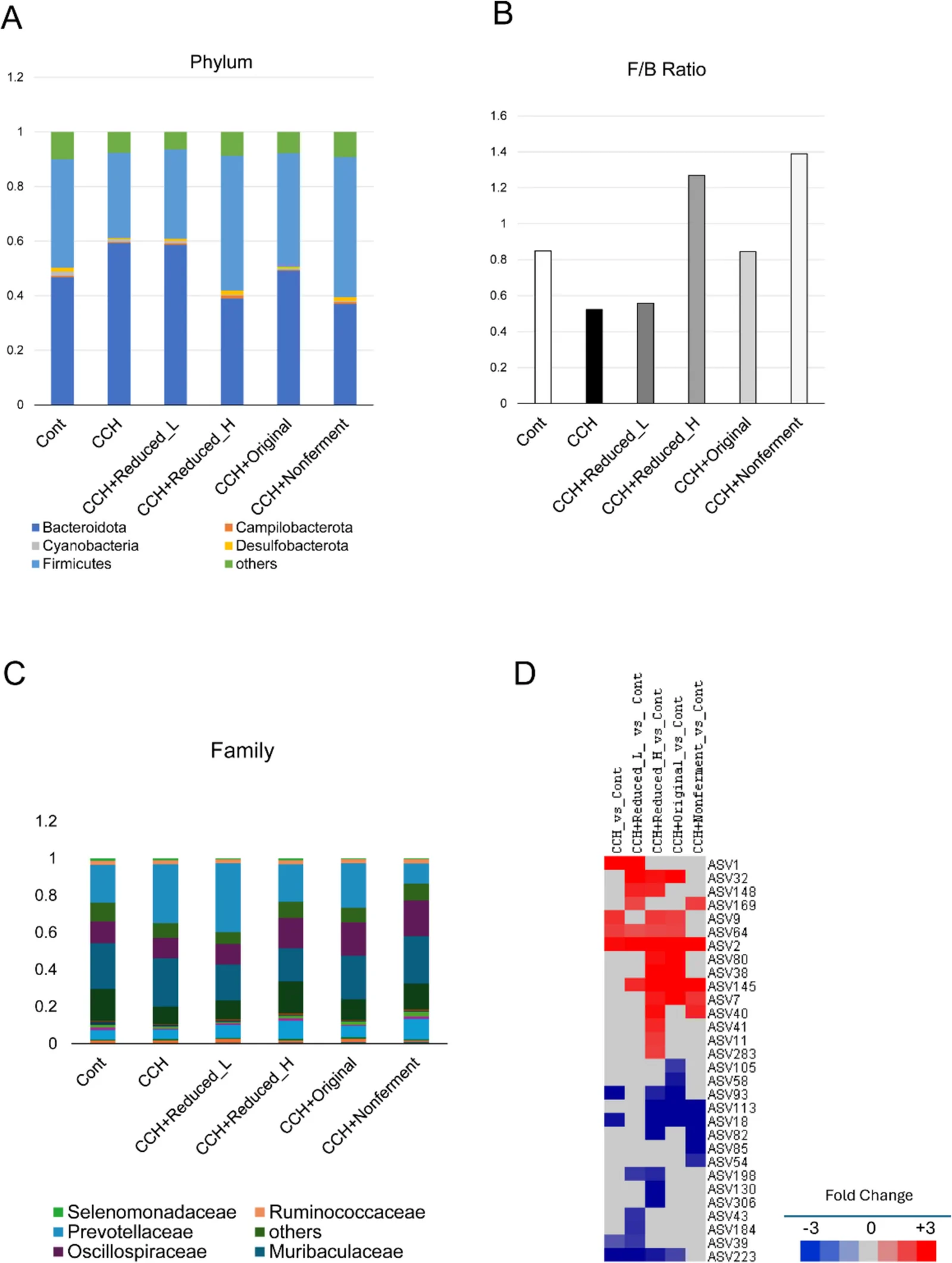
**The composition of gut microbiota after Hua-Feng-Dan treatment.** Bacterial DNA was isolated from the contents of the colon content and subjected to 16 S rRNA-seq. **A**. The major phyla levels of microbiota composition after Hua-Feng-Dan treatment. **B**. *Firmicutes/Bacteroidetes* (F/B) ratio after Hua-Feng-Dan treatment. C. Microbiota composition at the family level after Hua-Feng-Dan treatment. D. 2-D cluster of ASV abundance among groups after Hua-Feng-Dan treatment Red represents upregulation and blue represents downregulation. (*n* = 4)

The relative abundances of the five predominant bacterial phyla were quantified in rats. In comparison with the Cont group (46.7%), the CCH group exhibited an increase (59%) in *Bacteroidetes* abundance. In contrast, the relative abundances of *Bacteroidetes* in the CCH+Reduced_H, Original, and Nonferment groups decreased to 39.01%, 49.18%, and 36.94%, respectively. *Desulfobacterota* abundance was 1.41% in the Cont group, but decreased to 0.55% in the CCH group; conversely, it increased to 1.46%, 0.62%, and 1.44% in the CCH+Reduced_H, Original, and Nonferment groups, respectively (Fig. 8A). The *Firmicutes*-to-*Bacteroidetes* (F/B) ratio—a well-established indicator of gut microbial equilibrium, closely associated with host metabolic regulation, energy harvest, and intestinal homeostasis—was 0.85 in the Cont group. This ratio decreased to 0.52 in the CCH group and increased to 1.27, 0.84, and 1.39 in the Reduced_H, Original, and Nonferment groups, respectively (Fig. 8B).

The relative abundances of 9 major bacterial families in colonic microbiota of rats were quantified via 16 S rRNA-seq. As illustrated in Fig. 8C, compared with the Cont group, the CCH group exhibited a statistically significant increase in the relative abundances of *Acidaminococcaceae* and *Prevotellaceae*, concomitant with a significant decrease in *Desulfovibrionaceae* and *Lachnospiraceae*. Treatment with Reduced_H, Original, and Nonferment formulations of Hua-Feng-Dan effectively reversed these dysbiosis shifts; however, the Reduced_L intervention failed to produce a restoration. Notably, Hua-Feng-Dan administration across all treatment groups did not induce significant alterations in the relative abundances of *Bacteroidaceae*, *Christensenellaceae*, or *Clostridiaceae*.

ASV (Amplicon Sequence Variant) could detect bacterial DNA variation. Figure 8D shows ASV1 abundance increased significantly in both the CCH and CCH+Reduced_L groups, whereas CCH+Reduced_H groups, Original and Nonferment treatment restored ASV1 abundance to normal. ASV39 abundance decreased in both the CCH and CCH+Reduced_H groups, whereas no decrease was detected in the Original and Nonferment groups. ASV18 abundance exhibited consistent temporal or treatment-related trends across the Cont, Reduced_H, Original, and Nonferment treatment groups. Compared with the CCH group, the Reduced_H, Original, and Nonferment interventions attenuated the down-regulation of ASV223.

## Discussion

The present study confirmed cognition deficits and neuron damage in CCH rats (Ma et al., 2025). Hua-Feng-Dan recipes (Reduced, Original, and Nonferment) alleviated CCH-induced behavioral deficits, prevented neuron damage in hippocampal CA1 regions, while Reduced_L was ineffective. Hippocampal RNA-seq revealed differential gene expressions among groups. IPA revealed the top Canonical pathways and Upstream regulators related to inflammation, IGF and glucose metabolism, and ion transport were upregulated in CCH rats, which were attenuated by Hua-Feng-Dan recipes. RT-qPCR verified RNA-Seq findings. CCH-decreased Firmicutes/Bacteroidetes ratio were recovered by Hua-Feng-Dan recipes. CCH-increased dysbiosis were brought to normal by Hua-Feng-Dan recipes; but Reduced_L was ineffective in these Omics analysis. Thus, this study demonstrated the beneficial effects of Hua-Feng-Dan in the treatment of CCH-induced chronic cerebral ischemia and revealed major molecular targets associated with the protection.

Cognitive deficits are the major manifestation in CCH rats. MWM and NOR are the two most commonly used behavioral methods for assessing CCH cognitive function (Ma et al. 2025b). The escape latency and the number of new object explorations in the CCH rats were significantly reduced than those in the Cont group. Unilateral common carotid artery ligation is an effective method for establishing the CCH rat model (Yang et al. 2016). This study clearly showed that Hua-Feng-Dan improved learning and memory impairments in CCH rats. Hippocampal CA1 is the brain region important for spatial leaning behavior (Jeong and Singer 2022) and memory function (Pachicano et al. 2025). Histopathology further confirmed the protective effects of Hua-Feng-Dan on hippocampal CA1 neurons in CCH rats, paving the road for the further RNA-Seq analysis. Hippocampal RNA-seq showed gene expression patterns varied among groups and could further provide the molecular targets for Hua-Feng-Dan to ameliorate CCH-induced behavioral deficits.

Ingenuity pathway analysis (IPA) is a useful bioinformatic tool to analyze molecular events for Hua-Feng-Dan protection. In IPA Canonical pathways, CCH-increased pathways related to ion transport, IGF metabolism and inflammation were brought to normal by Hua-Feng-Dan recipes except for Reduced_L, coincide with behavioral tests. However, the downregulation of other 12 pathways by Hua-Feng-Dan was observed (Fig. 4A), their implication require further investigation. CCH also increased Upstream regulators related to inflammation and metabolism (Fig. 4B), consistent with previous studies on CCH (Cao et al. 2024). All of these changes were attenuated by Hua-Feng-Dan recipes except for Reduced_L, and verified by RT-qPCR (Fig. 5), indicating that the beneficial effect of Hua-Feng-Dan is closely related to anti-inflammatory properties (Hu et al. 2020; Chen et al. 2020; Han et al. 2025; Zhang et al. 2024).

Heatmap showed genes associated with ion channels and electrophysiology (e.g., Kcnj13, Kcne2, Clic6, Aqp1, SIc4a5,Slc13a4) and cellular structure (e.g., Cldn2, Mfrp, Vamp2) were increased, and 6 of them were verified by RT-qPCR (Fig. 6). For example, Aqp1 (aquaporin-1), a water channel protein, is associated with severe hypoxia-induced hydrocephalus and pronounced cognitive impairment (Trillo-Contreras et al. 2018; Liu et al. 2024b). Reduced Aqp1 after cerebral ischemia is associated with reuced blood-brain barrier damage and brain edema (Liu et al. 2024b). Under conditions of cellular hypoxia/reoxygenation, Cldn2 (claudin2) is upregulated (Wang et al. 2021b) and inhibiting the expression of Cldn2 is helpful in maintaining the blood-brain barrier under the CCH state (Fiorentino et al. 2016). Kcnj13 (potassium inwardly-rectifying channel, subfamily J, member 13) overexpression will increase inward K⁺ conduction and disrupt signal transmission (Judge et al. 2007) and the function of the hippocampus (Yan et al. 2026). Potassium channel KI is a nervous system therapeutic target (Judge et al. 2007). Mfrp (membrane frizzled-related protein) encodes a protein that modulates the Wnt signaling pathway(Kameya et al. 2002). Pnisr (proliferation-inducing and survival-regulating gene) regulates cell proliferation and survival (Liu et al. 2024c). These CCH-increased genes were attenuated by Hua-Feng-Dan recipes, and could contribute to the protection.

Microbiota-gut-brain axis plays an important role in the pathogenesis of CCH (Li et al. 2025). The cognitive impairment caused by CCH can be improved by adjusting the diversity and composition of gut microbiota (Song et al. 2024). Significant increases in *Bacteroidetes* levels, alongside marked reductions in metabolic regulation-related bacteria including *Firmicutes* were observed in cerebral ischemia models (Zhuang et al. 2025). We found that the levels of *Bacteroidetes* were elevated in CCH, accompanied by a decrease in *Firmicutes*, resulting in a decreased F/B ratio. Consistent with CCH models (Wang et al. 2021a; Ma et al. 2025a), and Hua-Feng-Dan reversed such decrease.

On the family level, CCH increased the relative abundances of *Prevotellaceae*, concomitant with a decrease in *Desulfovibrionaceae* and *Lachnospiraceae*. Previous studies have shown that *Prevotellaceae* plays an important role in vascular cognitive impairment (Liu et al. 2025) and in mouse MCAO model (Zhang et al. 2022). Hua-Feng-Dan treatment reversed these changes. The increased *Prevotellaceae* (phylum *Bacteroidetes*) is linked to gut inflammation (Elinav et al. 2011), suggesting that Hua-Feng-Dan might inhibit the inflammatory response that develops after CCH. *Lachnospiraceae* is a family of bacteria from the phylum *Firmicutes*. Decreased *Lachnospiraceae* after MCAO in mice could be reversed by Angong Niuhuang Pill (Zhang et al. 2022). Compared with the CCH group, the reduced abundance of *Lachnospiraceae* was also reversed after Hua-Feng-Dan treatments, consistent with the changes at the phylum level. Furthermore, we also observed that the abundance of *Desulfovibrionaceae* in CCH decreased. Hua-Feng-Dan can increase their abundance and thereby exert an anti-CCH dysbiosis effect.

ASV1 (a specific bacterial Amplicon Sequence Variant) is often identified as a member of the *Prevotellaceae* family, which plays significant roles in carbohydrate metabolism, gut health, and host-microbe interactions (Newman et al. 2024) The results indicate that the changes of ASV1 are consistent with the *Prevotellaceae* abundance. ASV39 is a specific, high-abundance bacterial sequence within the *Muribaculaceae* family. *Muribaculaceae* is a anti-inflammatory bacteria, the abundance of which was decreased in cerebral ischemia-reperfusion injury in mice (Wang et al. 2023). In this study, CCH-decreased ASV39 was recovered by Hua-Feng-Dan recipes.

Modulation of gut microbiota by Hua-Feng-Dan has been demonstrated to play roles against LPS + MPTP-induced neuroinflammation in mice (Hu et al. 2020), against LPS + Rotenone-induced Parkinson’s disease model in rats (Chen et al. 2020), against PTZ-induced convulsion in mice (Li et al. 2026), and against MCAO-induced acute cerebral ischemia in rats (He et al. 2025). This study clearly demonstrated the protection mechanism of Hua-Feng-Dan recipes are also linked to attenuation of CCH-induced dysbiosis.

It should also be mentioned that the therapeutic effect of Reduced_H, Original and Nonferment Hua-Feng-Dan were effective against CCH, although to different extents, while Reduced_L was ineffective in majority assays. Contrary to our initial hypothesis and expection from our prior in vitro (Zhang et al. 2012) and in vivo (Chen et al. 2020) studies on the nessasrity of including cinnabar (αHgS) and realgar (As4S4), the reduced_H recipe was still effective, despite less effective than Original in some parameter. Hua-feng-Dan“*yaomu*” plays important role in protecting MCAO-induced acute cerebral ischemia (Han et al. 2025), but Nonferment Hua-Feng-Dan is also effective, though to a less extent. These observations were quite similar to the anti-PTZ-induced seisures by Hua-Feng-Dan recipes (Li et al. 2026). The exact role of adding cinnabar, realgar, and 3-month fermentation, however, still needs further critical evaluation.

Limitations in the study include: (1) Sex may influence CCH-related cognitive and inflammatory responses (Towfighi et al. 2011). However, only male rats were used in this study, female animals will be considered in future studies; (2) the specific bioactive molecules of Hua-Feng-Dan responsible for protection against CCH require further investigation; (3) White matter pathology and BBB integrity, microglial/astrocytic activation and vascular pathology should be considered in future studies.

## Conclusion

This study clearly demonstrated that Hua-Feng-Dan recipes (Reduced_H, Original, and Nonferment) are effective against CCH-induced chronic cerebral ischemia, evidenced by behavioral tests, histopathology, hippocampal transcriptome profiling, and gut microbiota analysis, providing the experimental basis for clinical application of traditional medicine Hua-Feng-Dan in the treatment of chronic cerebral ischemia.

## Data Availability

The RNA-seq generated in the study are publicly available. The raw data can be found DOI:10.5281/zenodo.20719036.
